# Genetic analysis of 32 InDels in four ethnic minorities from Chinese Xinjiang

**DOI:** 10.1371/journal.pone.0250206

**Published:** 2021-04-22

**Authors:** Yujie Huang, Xiaoying Chen, Cong Liu, Xueli Han, Chao Xiao, Shaohua Yi, Daixin Huang

**Affiliations:** Department of Forensic Medicine, Tongji Medical College, Huazhong University of Science and Technology, Wuhan, Hubei Province, China; Xiamen University, UNITED STATES

## Abstract

The present study used the previously constructed 32-plex InDels panel to investigated the genetic diversity of four ethnic minorities (Hui, Mongol, Uygur and Kazakh) from Xinjiang, and analyzed the genetic relationships between the four populations and 27 reference populations. No significant deviations were observed from the Hardy-Weinberg equilibrium (HWE) at the 32 InDels for each population. The average observed heterozygosity (H_exp_), average polymorphic information content (PIC), combined power of discrimination (CPD) and cumulative probability of exclusion (CPE) for the 32 InDels were all higher than the Qiagen Investigator DIPplex kit in the four populations from Xinjiang. The CPD ranged from 0.999999999999903 (Kazakh) to 0.999999999999952 (Hui) and CPE ranged from 0.9971 (Uygur) to 0.9985 (Hui), which indicated that the 32 InDels were capable for individual identification and could be a supplementary tool in paternity test for these populations. Population genetic analysis by the method of analysis of molecular variance (AMOVA), *F*_*ST*_, phylogenetic tree, TreeMix-based topology, multi-dimensional scale analysis (MDS), principal components analysis (PCA) and STRUCTURE analysis showed that Xinjiang Hui population has a close relationship with East Asians (EAS), especially Chinese Han, and the populations of Xinjiang Mongol, Uygur and Kazakh showed mixed ancestral components related to EAS and Europeans (EUR).

## Introduction

Insertion/deletion polymorphisms (InDels) are biallelic genetic markers that widely distributed in the human genome with the average of one InDel per 7.2 kb [1], and have become one of the research hotspots in the field of forensic genetics in recent years. Comparing to short tandem repeats (STRs), InDels have the characteristics of smaller amplicons and lower mutation rate [2]. In addition, InDels are length polymorphic markers the same as STRs, which could be detected and analyzed by the commonly used forensic DNA testing platform. With these advantages, various human identification panels using InDels have been reported and put into forensic application. Among them, the Qiagen Investigator DIPplex^®^ kit (Qiagen, Hilden, Germany) has become the most widely used commercial InDel kit at present. Besides, the AGCU InDel 50 kit (AGCU ScienTech Inc., Wuxi, China) which contains 47 autosomal InDels, 2 Y-InDels and Amelogenin has been newly developed, and was widely used in population genetic studies in different Chinese ethnicities [3–5]. According to previous studies, InDels have been applied to individual identification of degraded DNA [6], human tumour tissues [7], unbalanced mixed stains [8], and ancestral inference [9, 10]. In our previous study, a 32-plex InDels panel was constructed and evaluated in Han populations from Hubei province, China [11]. The panel was considered to be highly polymorphic for Chinese Han, effective for degraded DNA, and was sensitive for low copy number (LCN) DNA.

Xinjiang, located in the hinterland of Eurasia and Northwest China, is the largest province in China and a multi-ethnic gathering area with 47 nationalities. Among these nationalities, 13 main ethnic groups, including Uygur, Han, Kazakh, Hui, Kirgiz, Mongol, and so on, have lived for generations with a long history. As of 2015, the total population of Xinjiang is 23.6 million. Uygur is the most populous ethnic group in Xinjiang, and the population is 11.33 million, accounting for 47.89% of the total population. Kazakh, Hui, and Mongol are also nationalities with large populations in Xinjiang, and both the Kazak and Hui populations are over one million, the Mongolian population exceeds 180,000. Historical research of the ethnic populations in Xinjiang showed complex population migration and ethnic blending during thousands of years, which led to genetic variation between different ethnic groups. Previous population genetic or genomic studies have demonstrated the extensive population admixture of Xinjiang populations related to western or eastern Eurasian. For example, study by Ning et al. showed that the Iron Age genomes from Xinjiang are highly structured with genetic ancestry related to both eastern and western Eurasians [12]. Genetic studies based on genome-wide DNA data [13] and Y chromosome [14] have shown that modern Uygurs are a mixed-race group mainly derived from eastern and western Eurasians. Besides, mtDNA [15] had also been used to reveal population differentiation and varied genetic admixtures among different ethnic groups in Xinjiang.

In the present study, we used the 32-plex InDels panel to evaluate the genetic diversity of the four ethnic minorities from Xinjiang including Hui, Mongol, Uygur and Kazakh. Furthermore, population genetic analysis was also conducted between the four populations and 27 worldwide reference populations, aiming to explore the genetic structure of the four Xinjiang ethnic minorities.

## Materials and methods

### Sample collection

This study was approved by the Medical Ethics Committee of Tongji Medical College, Huazhong University of Science and Technology (registration number: Tongji-2019-IEC-S160). Healthy adult volunteers residing in Xinjiang were recruited to provide blood samples through recruitment adverts during May-June 2019. All participants were interviewed to ensure that they do not have acute or chronic medical illnesses and their ancestors were of the same race going back at least three generations, and signed the written informed consent statements prior to sample collection. Blood was obtained by finger prick and collected using medical gauze. After air dried, the bloodstains were stored at −20°C until processed. Blood samples were collected from a total of 600 unrelated individuals from four ethnic minorities in Xinjiang in an anonymous way, including 150 Hui (64 males and 86 females), 150 Mongolians (77 males and 73 females), 150 Uygurs (80 males and 70 females) and 150 Kazakhs (76 males and 74 females).

The 27 reference populations used in the study consist of 26 global populations from the 1000 Genomes Project phase 3 (n = 2504) [16] and a Chinese Han population of Hubei province from our previous report (n = 204) [11]. The detailed information of the studied populations and reference populations was summarized in S1 Table. The geographic positions of the studied Xinjiang populations and the reference populations were shown in S1 Fig.

### DNA extraction, amplification and genotyping

Genomic DNA was extracted using the Chelex-100 method [17], and quantified by the Nanodrop 2000 spectrophotometer (Thermo Fisher Scientific, MA, USA). The multiplex PCR amplification of the 32 autosomal InDels were conducted according to the PCR protocol described in our previous research [11] on GeneAmp 2720 (Thermo Fisher Scientific, MA, USA). The PCR products were subsequently separated and detected via the ABI 3130 Genetic Analyzer (Thermo Fisher Scientific, MA, USA), and were analyzed by the GeneMapper ID v3.2 software (Thermo Fisher Scientific, MA, USA).

### Statistical analysis

Allele frequencies and forensic parameters including power of discrimination (PD), power of exclusion (PE) and polymorphic information content (PIC) were calculated using the spreadsheet PowerStats v1.2 [18]. The expected and observed heterozygosity (H_exp_ and H_obs_), exact test of Hardy-Weinberg equilibrium (HWE), linkage disequilibrium (LD), locus by locus AMOVA and pairwise *F*_*ST*_ [19] were performed and obtained using the Arlequin v3.5 software [20]. The *Nei’s D*_*A*_ distances between populations were obtained by the DISPAN program on the basis of allele frequencies. The heatmap of allele frequencies and *D*_*A*_ distance matrix was created by the R Statistical Software v3.6.2. The multi-dimensional scale analysis (MDS) (based on *D*_*A*_ distances) was conducted by the SPSS Software v20.0 (Chicago, Illinois) and the principal components analysis (PCA) (based on allele frequencies) of the total 31 populations was performed the Origin Software v2019b (Northampton, MA, USA). In addition, the phylogenetic tree by neighbor joining method [21] was constructed using the MEGA X Software [22] based on the *D*_*A*_ distances, the TreeMix-based topology was constructed using the graph-based program TreeMix version 1.13 [23], and STRUCTURE v2.3.4 [24] was employed to describe and visualize the population clustering using the genotype data of each individual.

## Results and discussion

### Genetic polymorphisms of 32 InDels in four ethnic minorities

Allele frequencies and forensic parameters of the 32 InDels in Xinjiang Hui, Mongol, Uygur and Kazakh were provided in S2 Table. The deletion frequencies varied from 0.3667 (rs25552) to 0.6967 (rs35149698) in Xinjiang Hui, from 0.3167 (rs3841948) to 0.7233 (rs34843628) in Xinjiang Mongol, from 0.3367 (rs2307561) to 0.7667 (rs34843628) in Xinjiang Uygur, and from 0.3267 (rs17515041) to 0.7267 (rs34843628) in Xinjiang Kazakh. Among the 32 InDels, there were only 4 loci (rs35149698, rs25552, rs2308189 and rs6481) with minimum allele frequencies (MAF) less than 0.4 in Xinjiang Hui, and the numbers were 9, 12 and 16 in Xinjiang Mongol, Uygur and Kazakh, respectively.

The 32 InDels in our study were distributed on 11 chromosomes, and the InDels on the same chromosome were more than 10 Mb apart to avoid linkage [11]. LD analyses for InDels on the same chromosome were carried out in the four Xinjiang populations separately, and there were totally 13 pairs of InDels tested. The result of the exact test showed no significant LDs between all the InDel pairs (significance level was 0.05/13 ≈ 0.0038 after Bonferroni correction), and thus the 32 InDels were independent from each other in heredity. HWE tests for the 32 InDels in the four populations showed no significant deviation after Bonferroni correction (significance level was 0.05/32 ≈ 0.00156) (see S2 Table).

The mean values of H_obs_, H_exp_, PIC, and the CPD, CPE in the 4 ethnic minorities in Xinjiang were listed in Table 1, and the forensic parameters of Xinjiang Hui, Uygur and Kazakh by the Investigator DIPplex kit was also listed for comparison [25–27]. The mean values of H_obs_ and H_exp_ ranged from 0.4706 (Xinjiang Uygur) to 0.4908 (Xinjiang Hui) and from 0.4766 (Xinjiang Kazakh) to 0.4918 (Xinjiang Hui), respectively. The CPD and CPE ranged from 0.999999999999903 (Xinjiang Kazakh) to 0.999999999999952 (Xinjiang Hui) and from 0.9971 (Xinjiang Uygur) to 0.9985 (Xinjiang Hui), respectively. Overall, Xinjiang Hui had the highest mean values of H_obs_, H_exp_, PIC, and the highest CPD and CPE among the four populations, which indicated that Xinjiang Hui has a relatively higher level of genetic polymorphism at the 32 InDels among the four Xinjiang ethnic groups. Although the CPD values were high enough for personal identification, the CPE values were all less than 0.9999 and thus this panel could not be used for paternity testing alone. Comparing with the Investigator DIPplex kit, the forensic parameters of Xinjiang Hui, Uygur and Kazakh for the 32-plex panel were all higher than the Investigator DIPplex kit except for the mean value of H_obs_ of Uygur (Table 1), which proved that the 32-plex InDels panel could become a valid and better alternative tool in forensic application for different nationalities in China.

**Table 1:** Forensic parameters of the four ethnic minorities from Xinjiang in our study and three reference populations by the Investigator DIPplex kit.

Populations		MH_obs_^a^	MH_exp_^b^	MPIC^c^	CPD^d^	CPE^e^
Our study	Xinjiang Hui	0.4908	0.4918	0.3699	0.999999999999952	0.9985
	Xinjiang Uygur	0.4706	0.4789	0.3630	0.999999999999937	0.9971
	Xinjiang Kazakh	0.4777	0.4766	0.3619	0.999999999999903	0.9977
	Xinjiang Mongol	0.4858	0.4849	0.3663	0.999999999999939	0.9981
Reference populations	Xinjiang Hui [25]	0.4239	0.4283	0.3330	0.99999999999378	0.9888
	Xinjiang Uygur [26]	0.4748	0.4781	0.3616	0.99999999999940	0.9963
	Xinjiang Kazakh [27]	0.4750	0.4680	0.3578	0.99999999999857	0.9973

^a^Mean value of observed heterozygosity.

^b^Mean value of expected heterozygosity.

^c^Mean value of polymorphic information content.

^d^Combined power of discrimination.

^e^Cumulative probability of exclusion.

### Interpopulation differentiation analysis among the 31 populations

**Fig 1:**
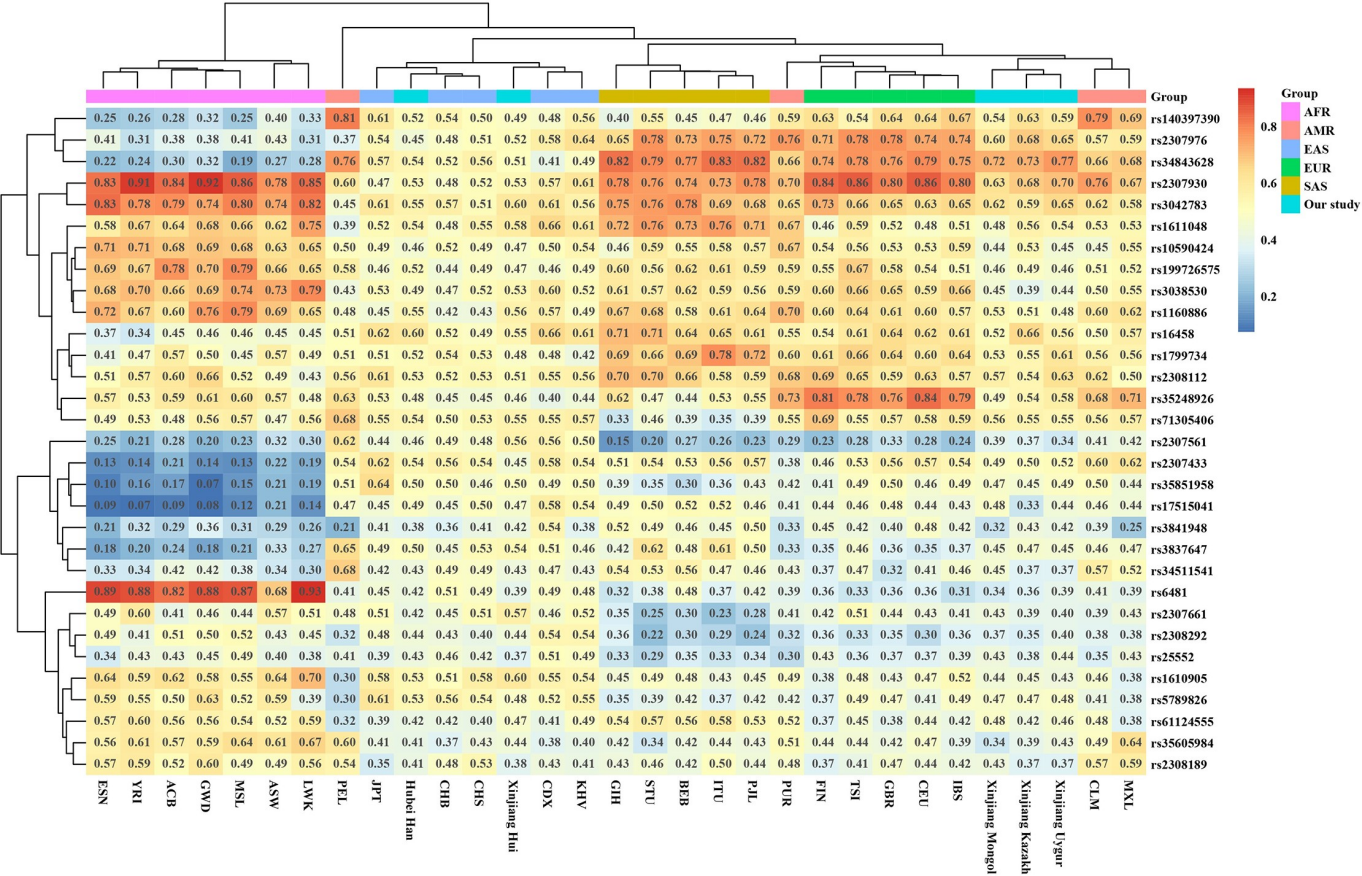
Heatmap of the 31 populations based on deletion frequencies of the 31 InDels in our study.

In order to show the frequency diversity of the InDels in our study among different populations around the world, the heatmap was conducted basing on the deletion frequencies of 31 populations (Fig 1), including 26 global populations from the 1000 Genomes Project phase 3 (2504 individuals in total, detailed frequency data in S3 Table), Chinese Han in Hubei (from our previous report [11]) and the 4 ethnic minorities from Xinjiang in this study. Among the 32 InDels, the data of rs35149698 were not given in the 1000 Genomes Project and this locus was therefore excluded in the heatmap and all the following population analysis. In addition, cluster analysis for the populations and InDels was carried out and displayed in the heatmap.

The 26 reference populations were classified into 5 groups according to geographical distribution: AFR (African), AMR (American), EAS (East Asian), EUR (European) and SAS (South Asian). The 4 Xinjiang populations, as well as Hubei Han, were grouped separately. It could be observed from the heatmap that populations from AFR, EAS, EUR and SAS were gathered in 4 main clusters, consistent with their intercontinental location. The 4 populations from AMR were not clustered, which probably because the American populations selected from the 1000 Genome Project are not native Americans and have different origins [28–30]. The similar result for Americans also appeared in the following analysis methods. Xinjiang Hui and Hubei Han populations were clustered with the EAS group, and the populations of Xinjiang Mongol, Uygur and Kazakh were clustered in a separate branch but near EUR. The cluster patterns revealed that Xinjiang Hui was closely related with EAS, especially with several Chinese populations, while Xinjiang Mongol, Uygur and Kazakh displayed close relationship with EUR group. According to previous studies, Xinjiang Hui had close genetic relationships with Chinese Han populations [25]. Xinjiang Uygur and Kazakh showed admixed genetic structure related to western or eastern Eurasian based on genome-wide DNA data and InDels [13, 27]. The results in our study were consistent with these previous reports. The deletion frequencies of the InDels in EAS were around 0.5, but had wider ranges in AFR, EUR and SAS, which means that our panel was highly polymorphic for East Asians.

As shown in Fig 1, rs2307976 and rs34843628 showed good discrimination ability in different continents. The deletion frequencies of rs2307976 were ≤ 0.43 in ARF, 0.45–0.64 in EAS, and 0.65–0.78 in EUR and SAS, and the frequencies of rs34843628 were ≤ 0.32 in ARF, 0.41–0.57 in EAS, and 0.74–0.82 in EUR and SAS. The results indicated that rs2307976 and rs34843628 could be considered as choices of ancestral information loci for future research. Rs2307930 and rs3042783 showed high deletion frequencies in AFR, EUR and SAS, while rs2307561 showed low deletion frequencies in AFR, EUR and SAS comparing to EAS. Besides, AFR populations had obviously higher deletion frequencies than other continents on rs6481, rs10590424, rs199726575, rs3038530 and rs116088, and lower deletion frequencies on rs2307433, rs35851958, rs17515041, rs3841948, rs3837647 and rs3451154. In our study, the deletion frequencies of rs3837647, rs35248926, rs3038530 and rs199726575 in four ethnic minorities of Xinjiang were similar to those of EAS, and the deletion frequencies of rs6481, rs34843628, rs5789826 and rs2308292 in Mongol, Uygur and Kazakh populations were similar to those of EUR except for rs34843628 and rs2308292 in Hui (similar to those of EAS). The deletion frequencies of rs2307976, rs2307561 and rs2307930 were between those of EUR and EAS except for rs2307561 and rs2307930 in Hui (similar to those of EAS).

The locus by locus AMOVA analysis between the four Xinjiang populations and the five continents was performed to compare the genetic differentiation (significant level at 0.05/31 = 0.0016). Xinjiang Hui, Xinjiang Mongol, Xinjiang Kazakh and Xinjiang Uygur all have the most significant differentiations with AFR at 23, 20, 25 and 24 loci, respectively. On the contrary, the minimum numbers of significantly different locus were 2 between Xinjiang Kazakh and EUR, and 3 between Xinjiang Uygur and EUR. There were only 2 loci observed significant differences between Xinjiang Hui and EAS, and 5 loci between Xinjiang Mongol and EAS or EUR. According to the results, the four populations from Xinjiang have the largest genetic differences with AFR, Xinjiang Hui has a close genetic relationship with EAS, Xinjiang Mongol was closest to EAS and EUR, and Xinjiang Kazakh and Xinjiang Uygur both appeared to have the closest relationship with EUR.

Pairwise *F*_*ST*_ and the corresponding *p* values (significant level at 0.0001 after Bonferroni correction) were calculated basing on the genotypes from our study and the reference populations (see S4 Table). The lower value of *F*_*ST*_ represented the less genetic differentiation between the two populations. Comparing with the other 30 populations, Xinjiang Kazakh had the lowest *F*_*ST*_ value (0.0012) with Xinjiang Uygur, and the two populations did not show significant difference at *p* = 0.0901. Xinjiang Hui had the lowest *F*_*ST*_ value (0.0020) with CHS (Southern Han Chinese), and did not show significant difference with CHS and Hubei Han at *p* = 0.0451 and 0.009. Xinjiang Mongol had the lowest *F*_*ST*_ value (0.0019) with Xinjiang Uygur, and was not significantly different with Xinjiang Uygur at *p* = 0.0451. The lowest *F*_*ST*_ value for Hubei Han was 0.0002 with CHB (Chinese Han in Beijing), and was not significantly different with Xinjiang Hui (*p* = 0.0090), CHB (*p* = 0.3243), CHS (*p* = 0.2973) and JPT (Japanese in Tokyo, *p* = 0.0180). The remaining pairwise comparisons showed significant population differentiations (*p* < 0.0001).

On the whole, the locus by locus AMOVA and pairwise *F*_*ST*_ revealed similar population genetic relationships with the heatmap.

### Genetic distance and phylogenetic reconstruction

The *D*_*A*_ distance is one of the methods to measure the genetic distance on the bases of genotype data, and using *D*_*A*_ distance to construct the phylogenetic tree could obtain relatively accurate results according to Nei et al. [30].

Pairwise genetic distances among the 4 Xinjiang populations and other 27 reference populations were calculated and represented using the parameter *D*_*A*_ (see S5 Table). For populations in our study, the minimum *D*_*A*_ values were 0.0011 between Xinjiang Kazakh and Xinjiang Uygur, followed by 0.0012 (between Hubei Han and CHB/CHS) and 0.00013 (Xinjiang Hui and Hubei Han, Xinjiang Uygur and Xinjiang Mongol). Besides, Xinjiang Kazakh, Uygur, Mongol and Hui all had the closest genetic distance with most EAS populations, and had the farthest distance with ESN (Esan in Nigeria, AFR). From the perspective of the five continents, the intracontinental *D*_*A*_ values were all less than 0.0059 in AFR, EAS, EUR and SAS, however, the *D*_*A*_ values between populations within AMR were large with the range of 0.0023 (MXL and CLM) to 0.0159 (PUR and PEL). For intercontinental genetic distance, populations from AFR showed the largest genetic differentiation with other continents (0.0127 ≤ *D*_*A*_ ≤ 0.0471).

**Fig 2:**
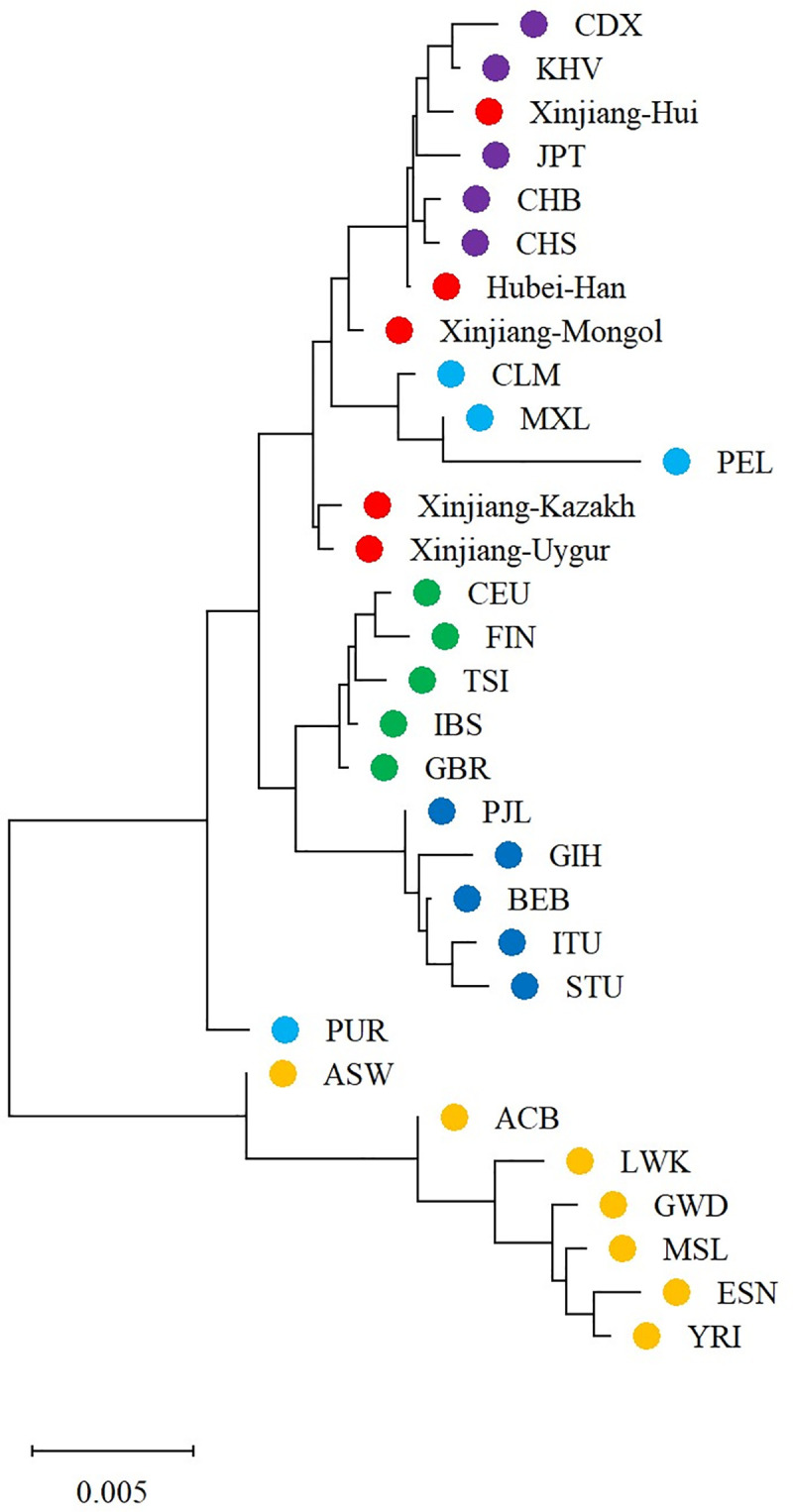
Phylogenetic tree (neighbor-joining tree) of the four studied Xinjiang populations, Hubei Han, and other 27 reference populations. Each dot denoted a population and different colors denoted different geographical regions: yellow for African, light blue for American, purple for East Asian, green for European, deep blue for South Asian, red for the four studied Xinjiang populations and Hubei Han.

The phylogenetic tree (neighbor-joining tree) was constructed basing on the *D*_*A*_ distances to reveal the genetic relationships of the 31 populations (Fig 2). It could be observed that populations of AFR were classified into one main branch and EAS, AMR, EUR and SAS were in another. Populations of each continent were grouped together except AMR (PUR in a separate branch and the other three clustered in EAS). Similar to Hubei Han, Xinjiang Hui was clustered with EAS, which might due to the close ethnic communication between Han and Hui populations in history. Xinjiang Mongol first clustered with Xinjiang Hui, Hubei Han and EAS, and then clustered with Xinjiang Kazakh and Xinjiang Uygur.

### TreeMix analysis

To investigate the genomic admixture (migration) between different populations, the TreeMix-based topology was constructed using the graph-based program TreeMix version 1.13 [23]. Applying the covariance of the allele frequency profiles as input, we ran TreeMix v.1.13 with migration events (m) varying from 0 to 5 to generate the topology with the maximum likelihood. The final analysis was performed with the optimal gene exchange route model at m = 2, with default settings used for all other parameters.

**Fig 3:**
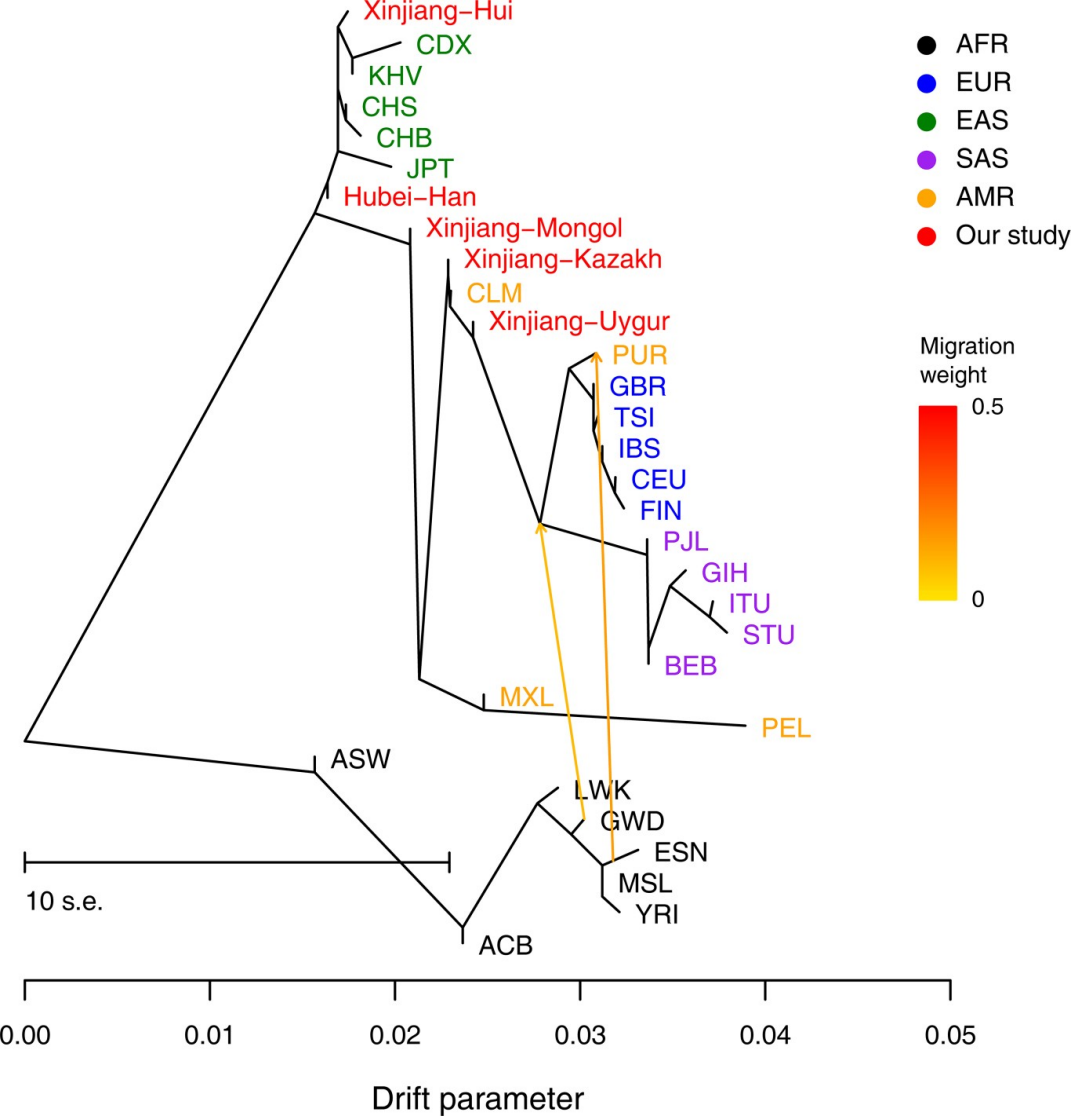
The maximum likelihood tree showed the patterns of population splits and genetic admixture with two migration event. The YRI, ESN, MSL, GWD, LWK, ACB and ASW samples were set as the outgroup. Horizontal branch lengths are proportional to the amount of genetic drift that has occurred on the branch. The scale bar shows ten times the average standard error of the entries in the sample covariance matrix. The residual fit from this graph is shown in S2 Fig.

As shown in Fig 3, clustered patterns similar to the phylogenetic tree (Fig 2) were identified by the TreeMix analysis. For the studied five Chinese populations, Hubei Han and Xinjiang Hui was clustered with EAS, and Xinjiang Mongol, Uygur and Kazakh showed mixed ancestral components from EAS and EUR. However, no obvious gene flow events into the studied Chinese populations or from the studied Chinese populations into EUR or other EAS were identified.

### MDS and PCA analysis

**Fig 4:**
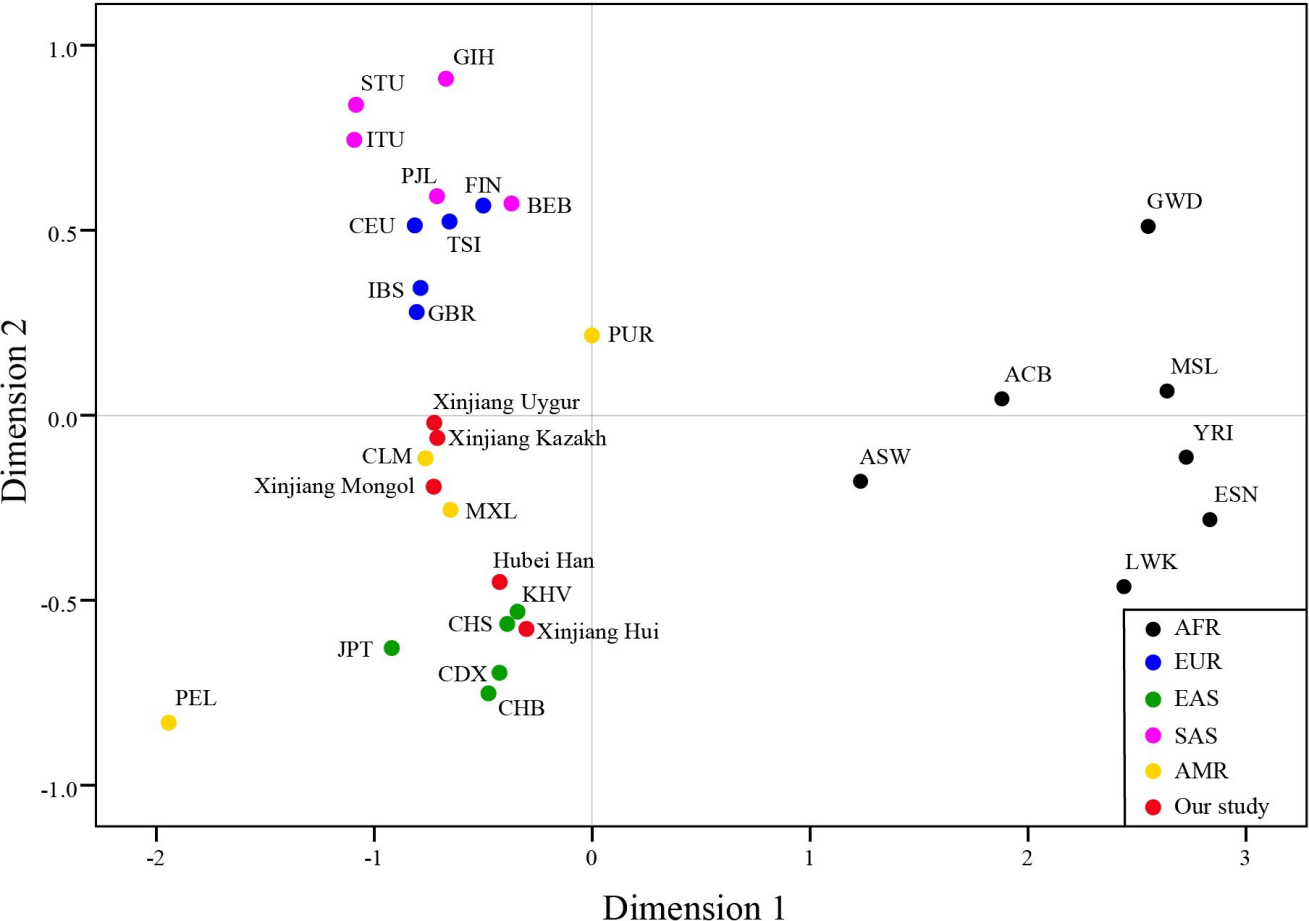
Multi-dimensional scale analysis of the 31 populations based on *D*_*A*_ distances.

As is shown in Fig 4, EAS and AFR could be distinguished from the other three continents, while several populations of EUR and SAS was very close in distance. The four populations from AMR were scattered between EUR and EAS. Xinjiang Hui and Hubei Han had close distances with East Asians, and Xinjiang Kazakh, Uygur, and Mongol were distributed between EUR and EAS, and were close with MXL and CLM from AMR.

**Fig 5:**
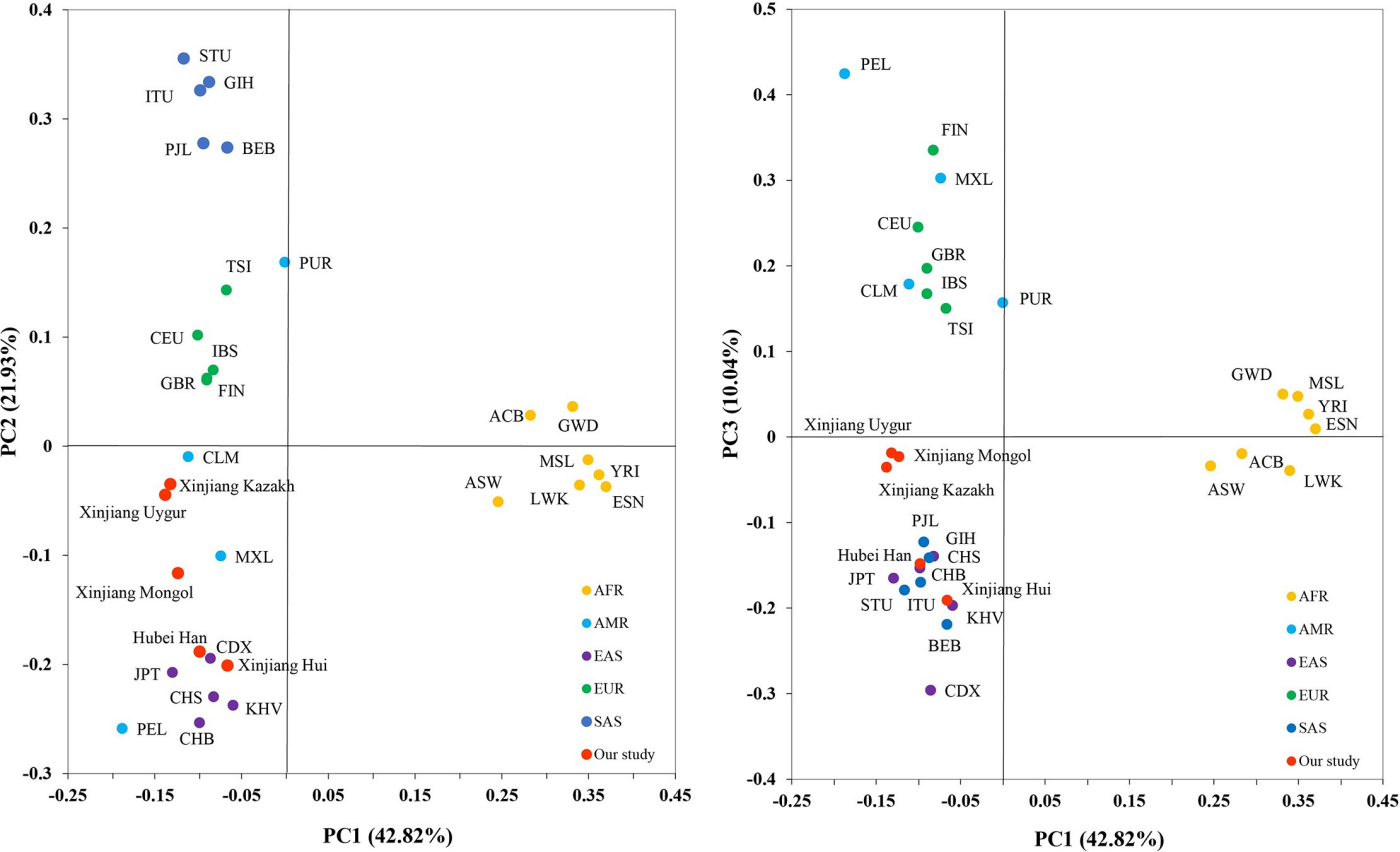
Principal component analysis of the 31 populations based on allele frequencies of the 31 InDels. PC1 and PC2 account for 64.75% of the total variation, and PC3 account for 10.04% of the total variation.

PCA analysis were conducted on the bases of allele frequencies of the 31 populations (Fig 5). The first three principle components (PC) contributed cumulatively 75.79% of the variation. PC1 (42.82% of the variation) mainly separated AFR from the rest of the populations. PC2 (21.93% of the variation) distinguishes EAS and SAS. PC3 (10.04% of the variation) separated AMR from EAS and could better distinguish SAS and EUR. Among the 5 continents, AMR was the only one that could not be separated from other continents. Xinjiang Hui and Hubei Han clustered with the EAS group. Xinjiang Kazakh, Xinjiang Uygur were close to each other, but did not clustered with any of the continents. Xinjiang Mongol was adjacent to Kazakh and Uygur in Fig 5 (right) but could be distinguished from the two populations by PC2 in Fig 5 (left).

The result of MDS and PCA analysis were generally consistent with the phylogenetic tree, and the PCA plot could better distinguish different continents than MDS.

### STRUCTURE analysis

**Fig 6:**
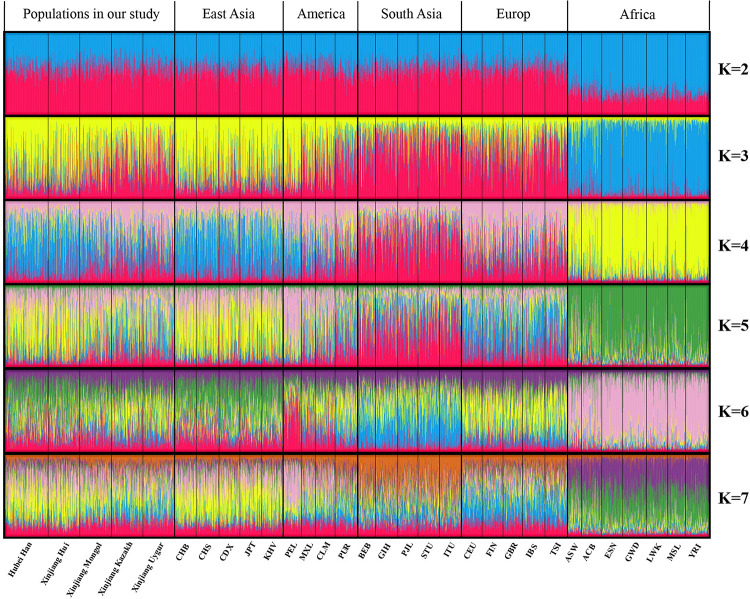
STRUCTURE analysis of the 31 populations based on the genotype data of each individual.

The STRUCTURE analysis can visually display the ancestral composition of different populations. Similar proportion of ancestral components reflects similar genetic background and ancestry origin among different populations. The STRUCTURE analysis result of the 31 populations for K = 2–7 was shown in Fig 6. According to the repeated sampling result by the CLUMPP program and Structure harvester (http://taylor0.biology.ucla.edu/structureHarvester/), the Delta K maximises at K = 3 and thus the optimal K value was estimated to be K = 3 [31]. When K = 2, only AFR could be distinguished from the rest populations. At K = 3, the 31 populations could be divided into 4 structure types: EAS (mainly in yellow); AFR (mainly in blue); EUR and SAS (mainly in red); Xinjiang Mongol, Kazakh, Uygur, and AMR (mixture of yellow and red). At K = 4–7, EUR could be distinguished from SAS by different ancestral components, and the genetic structure of Xinjiang Mongol, Kazakh and Uygur were similar with EUR. The ancestry proportions of Hubei Han and Xinjiang Hui were similar with EAS at K = 2–7. These results were consistent with the locus by locus AMOVA analysis, PCA, and phylogenetic tree.

Since there is no data of rs35149698 in the 1000 Genomes Project, we only compared the forensic parameters of 31 InDels between Xinjiang west-east mixed populations and EAS or EUR. As is shown in S6 Table, the MH_obs_, MH_exp_, MPIC, CPD and CPE of these 31 InDels in Xinjiang Hui are similar to those of EAS, which is higher than those of EUR, and these forensic parameters in Xinjiang Mongol, Uygur and Kazakh were all between those of EAS and EUR. These results indicated that the forensic application value of this InDels panel in EAS is higher than that in EUR. Overall, the population genetic analysis based on this InDels panel showed that Xinjiang Hui population has a close relationship with EAS, and the populations of Xinjiang Mongol, Uygur and Kazakh showed mixed ancestral components related to EAS and EUR, which is consistent with those previous reports [12−14, 25, 27].

According to historical research, the Xinjiang Hui population mainly originated from the large-scale migrations from northwest China since Qing Dynasty, and had close communication with Chinese Han during the migrations. Xinjiang Mongol, Uygur and Kazakh unceasing migrated and integrated with neighboring nationalities of Central Asia and Europe in the long-term historical process. In addition, according to the surveys in Xinjiang province, the intermarriage rate between Hui and Han, Uygur and Kazakh were relatively high, but was low between Han and Uygur [32]. These historical and cultural factors furthermore proved that Xinjiang Hui was genetically close to Chinese Han and EAS, while Xinjiang Mongol, Uygur and Kazakh showed mixed ancestral components of EUR and EAS. The conclusions were also consistent with previous studies of Xinjiang Hui [25], Uygur and Kazakh [27, 33].

## Conclusion

In this study, we obtained the genetic data of four ethnic minorities in Xinjiang including Hui, Mongol, Uygur and Kazakh at 32 InDels using the 32-plex InDels panel. It resulted that our panel was highly polymorphic and showed higher CPDs and CPEs compared with the Investigator DIPplex kit in all those Xinjiang populations, and so it could play an important role in forensic application. Population genetic analysis showed that these InDels could distinguish populations from EAS, EUR, SAS and AFR. Moreover, the result suggested that Xinjiang Hui had close genetic relationship with East Asians, especially Chinese Han, and the populations of Xinjiang Mongol, Xinjiang Uygur and Xinjiang Kazakh showed mixed ancestral components from EAS and EUR.

## Supporting information

S1 Fig The geographic positions of the studied Xinjiang populations and the reference populations. (A) the studied populations in Xinjiang and a reference Chinese Han population in Hubei. (B) 26 reference populations from the 1000 Genomes Project (phase 3).
(TIF)

S2 Fig Residual fit from graph of human population data presented in the main text. Plotted are the residuals from the fit of the graph presented in Fig 3 in the main text.
(TIF)

S1 Table Reference population samples and 4 studied population samples. (XLSX)

S2 Table Allele frequencies and forensic parameters of the 32 InDels in the four ethnic minorities from Chinese Xinjiang. MAF: minimum allele frequency; Hobs: observed heterozygosity; Hexp: expected heterozygosity; PD: power of discrimination; PE: power of exclusion; PIC: polymorphic information content; HWE-p: Hardy-Weinberg equilibrium p values.
(XLSX)

S3 Table Deletion frequencies of the 31 InDels (except for rs35149698) in the 26 reference populations from the 1000 Genomes Project (phase 3). (XLSX)

S4 Table Population pairwise *F_ST_* between the 31 populations. (XLSX)

S5 Table Genetic distance matrix of *D_A_* among the 31 populations. (XLSX)

S6 Table Forensic parameters of the 31 InDels in the EUR, EAS, Hubei Han and four ethnic minorities from Chinese Xinjiang. Hobs: observed heterozygosity; Hexp: expected heterozygosity; PD: power of discrimination; PE: power of exclusion; PIC: polymorphic information content.
(XLSX)
