# Supplementary figures and images for: Genetic analysis of 32 InDels in four ethnic minorities from Chinese Xinjiang
Source: PLoS One. 2021 Apr 22;16(4):e0250206. doi: 10.1371/journal.pone.0250206 (PMC8061914; doi:10.1371/journal.pone.0250206)

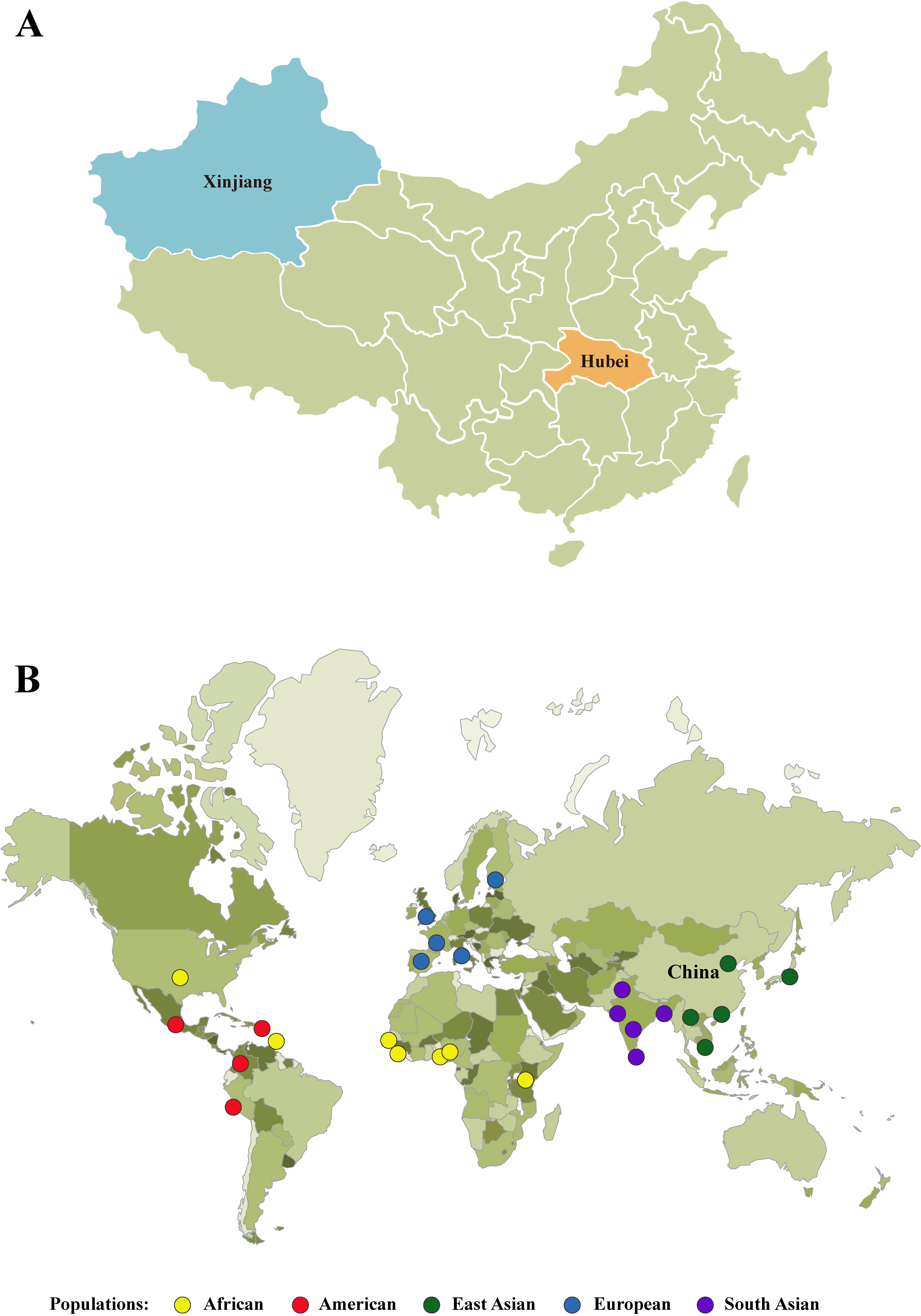

Supplement: S1 Fig — (A) the studied populations in Xinjiang and a reference Chinese Han population in Hubei. (B) 26 reference populations from the 1000 Genomes Project (phase 3). (TIF) [file pone.0250206.s001.tif]

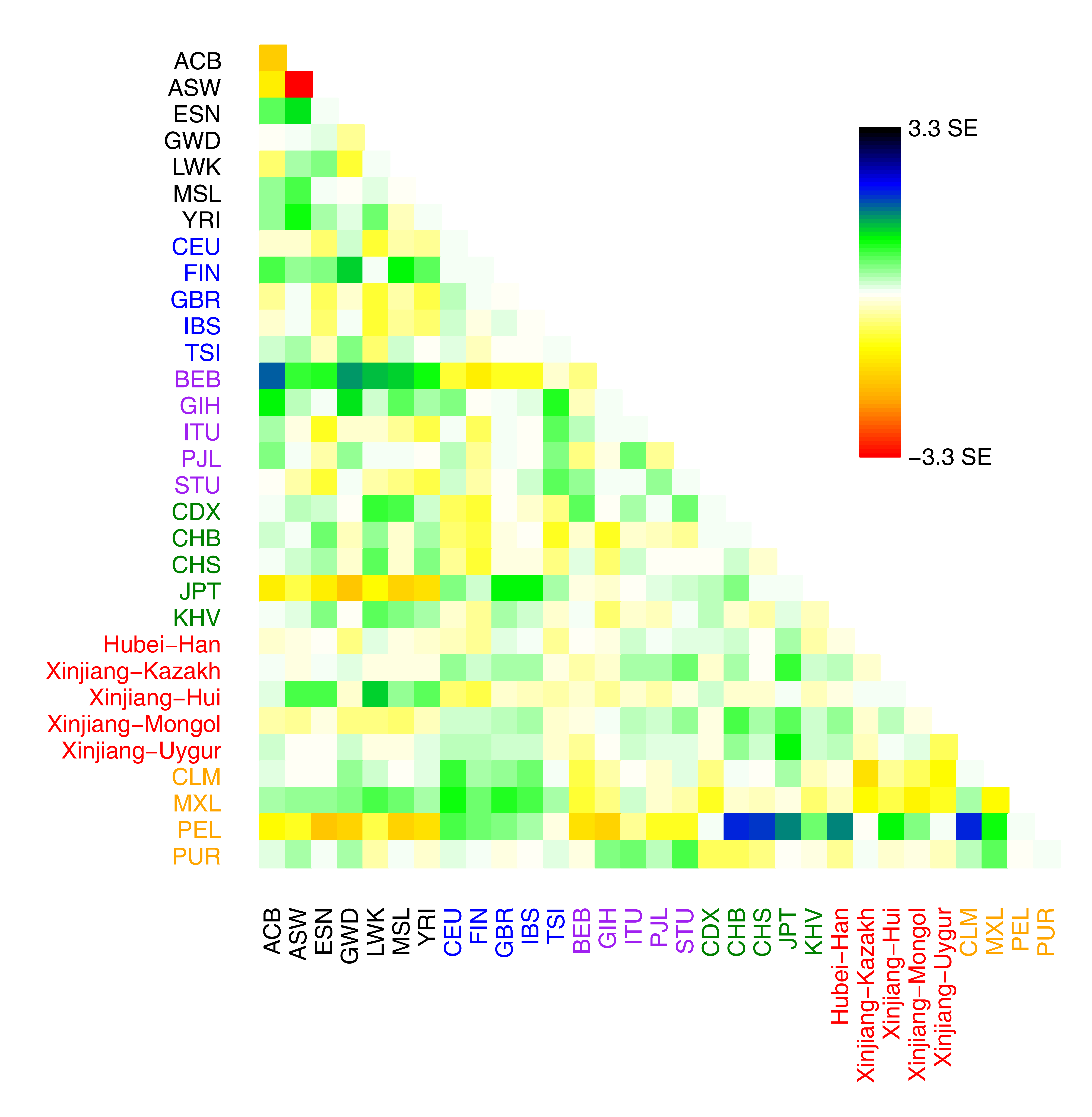

Supplement: S2 Fig — Plotted are the residuals from the fit of the graph presented in Fig 3 in the main text. (TIF) [file pone.0250206.s002.tif]
